# Beyond tool use: how ecological coupling configures AI’s empowerment for language learning engagement

**DOI:** 10.3389/fpsyg.2026.1747355

**Published:** 2026-01-22

**Authors:** Lingya Ge, Yun Xia, Chengfang Yang

**Affiliations:** 1 College of Chinese Language and Literature, Qufu Normal University, Qufu, China; 2 School of Foreign Languages, Xuzhou University of Technology, Xuzhou, China; 3 School of Translation Studies, Qufu Normal University, Rizhao, China; 4 College of Environmental Engineering, Xuzhou University of Technology, Xuzhou, China

**Keywords:** AI-empowerment Configural Model, artificial intelligence, ecological affordance theory, ecological coupling, ecological systems theory, language learning engagement

## Abstract

This study develops and tests an AI-empowerment Configural Model to explain how artificial intelligence (AI) empowers language learning engagement. Grounded in ecological systems theory (EST) and ecological affordance theory (EAT), the model theorizes AI as an interactive agent within the learning ecosystem. A mixed-methods study of 475 Chinese university language learners demonstrates that AI’S effect on engagement is significantly mediated by the perceived quality of its ecological coupling with teachers, peers, and the environment. Latent profile analysis (LPA) further identifies three distinct learner configurations: low coupling-low engagement, moderate coupling-moderate engagement and high coupling-high engagement, which systematically differ in their coupling of AI. The model ultimately shifts the paradigm from tool implementation to strategic ecological governance, providing a practical basis for designing learning environments that leverage synergistic human–AI coupling to foster deeper, sustained engagement.

## 1 Introduction

Artificial Intelligence (AI) is fundamentally reshaping the ecological landscape of language learning in higher education, unlocking unprecedented opportunities to enhance learning engagement, which is a well-established determinant of academic success in college English instruction. Yet, this potential remains underexplored. Prevailing research has predominantly centered on evaluating the effectiveness of discrete AI technologies. This is evident in studies that measure AI’s efficacy in assessing and alleviating public speaking anxiety (Zheng et al., 2024; Chen, 2024), its utility in providing feedback on non-verbal communication (Isotalus et al., 2024; Zeng et al., 2022), and its application in commercial tools for skill development (Mei et al., 2024; Shafiee Rad, 2024). Further examples include research on AI’s role in enhancing the efficiency and quality of slide design (Camp and Johnson, 2025). The prevailing focus on efficacy in existing research has overlooked a critical perspective: conceptualizing AI as an ecological agent that engages in systemic interactions within the learning ecosystem. This oversight has resulted in the absence of a theoretical framework capable of explaining how AI, through these interactions, shapes language learning engagement. To address this gap, this study proposes and investigates the core construct of “ecological coupling.” We posit that the key to understanding AI’s impact lies in the dynamic, synergistic quality of its coupling with other core elements of the learning microsystem, namely teachers, peers, and the environment. This research aims to (1) examine how the quality of this coupling mediates AI’s influence on engagement, and (2) identify distinct configurational patterns of learner experiences within this coupled ecosystem. The ultimate goal is to synthesize these insights into an AI-empowerment Configural Model, moving beyond explanatory theory to offer educators an actionable roadmap for designing more effective, AI-integrated language learning environments.

### 1.1 Theoretical background and conceptual framework

Artificial Intelligence (AI) is widely recognized as a central driver in reshaping foreign language education (Zheng, 2024), a trend exemplified by extensive practical explorations in areas such as AI-assisted language generation, multimodal instruction, and intelligent feedback systems (Yang, 2024; Zeng et al., 2023; Qin et al., 2025; Wen and Liang, 2024; Wei and Li, 2023). Despite this burgeoning activity, mainstream research remains predominantly anchored in a tool-centric paradigm. This paradigm narrowly evaluates AI’s direct, functional utility, such as enhancing instructional efficiency, personalizing tasks, or boosting engagement metrics (Huang et al., 2023; Almusaed et al., 2023; Wang and Xue, 2024) by treating it as an isolated technological intervention. Consequently, it overlooks AI’s embedded role as a constitutive element within the broader learning ecosystem.

Recent scholarship has begun to transcend this linear, input–output view by framing AI as a dynamic actor or interactant. This emerging perspective recognizes that AI’s utility is not intrinsic but is mediated by learners’ psychological needs and beliefs (Wang and Reynolds, 2024; Wang and Wang, 2024; Wang et al., 2025) and shaped by teachers’ perceptions of technology integration (Huang et al., 2025). This marks a critical shift from viewing AI as a standalone tool to conceptualizing it as a contextually embedded agent.

However, a critical and systematic theoretical gap persists. First, while acknowledging context, existing studies often examine individual factors in isolation, failing to systematically model the synergistic, multi-way interactions between AI and the core ecological elements such as teachers, peers, and the learning environment as an integrated system. Second, the literature frequently frames AI’s impact through a dualistic lens of empowerment vs. disempowerment, attributing outcomes to moderating variables like learner motivation (Xun et al., 2025; Huang et al., 2023). Yet, it lacks a robust explanatory mechanism to account for the heterogeneous and often contradictory outcomes observed in practice (Karataş et al., 2024; Shi and Aryadoust, 2024). Ultimately, even some ecological models still position AI as an external variable acting upon the system, rather than an integral agent co-evolving within it (Jiang, X., 2025). This creates a fundamental theoretical disconnect: although AI’s effects are widely acknowledged as context-dependent, we lack a coherent framework to explain how its specific mode of integration, namely its coupling with particular ecological elements, co-produces these varied effects on engagement.

To bridge this gap, our study is grounded in two key theoretical and operational foundations.

First, we adopt a comprehensive, four-dimensional framework of learning engagement, integrating behavioral, cognitive, and emotional dimensions with the critical addition of social engagement (Fredricks et al., 2004; Philp and Duchesne, 2016). Social engagement is operationalized as the quality and frequency of students’ interactions with teachers and peers (Xu et al., 2025; Xu and Yang, 2024; Xu and Long, 2022; Saeed and Alharbi, 2023), serving as a direct indicator for the key human agents within our ecological model.

Second, we integrate Ecological Systems Theory (EST) and Ecological Affordance Theory (EAT) to form our core analytical lens. EST provides the macro-structure, positing that development arises from interactions within nested systems (Bronfenbrenner, 1979; Bronfenbrenner and Morris, 2006). Within this structure, we reconceptualize AI as an active ecological agent within the microsystem, capable of initiating interactions and reshaping affordances, which is a perspective echoed by some scholars (Bian et al., 2025; Qin and Xu, 2025; Qin et al., 2021; Qin and Dai, 2013). The core of our empirical inquiry lies in the mesosystem, examining the relational configurations formed between AI and other elements (teachers, peers, environment).

EAT provides the micro-mechanism. Affordances are potentialities for action that emerge from the interaction between an agent’s capabilities and a learner’s perception within a specific context (Gibson, 1979; Chen, 2022; Tai et al., 2022). The realization of an AI affordance depends on the learner’s perception and is further shaped by interactions with teachers and peers as well as the environment (Xu and Yang, 2025). The perception-interpretation-action cycle provides a fundamental ecological mechanism for understanding how language learning engagement is dynamically realized (Van Lier, 2004; Qin and Xu, 2025), but is itself moderated by the quality of AI’s coupling with the broader ecology. These theoretical insights are critical for interpreting the dual and often contradictory nature of AI’s ecological affordances reported in recent studies. While AI is noted for reshaping cognitive strategies and interaction patterns, concurrent research highlights challenges such as inhibited deep negotiation (Michel et al., 2025) and knowledge fragmentation (Zhang, 2025). We argue that this duality is not inherent to AI itself but arises from variations in the quality of ecological coupling, which moderates how affordances are perceived and actualized.

### 1.2 Ecological coupling: the core construct

To bridge precisely the macro–micro disconnect, we propose ecological coupling as our core construct, serving as the conceptual bridge that integrates EST and EAT. Grounded in Gibson’s (1979) ecological psychology and advanced by Van Lier (2004) in language education, coupling refers to the dynamic, unified system formed through the continuous perception and utilization of affordances between an organism and its environment.

We posit that in an era where AI profoundly reshapes learning ecology, this classical concept must be extended to account for AI-human synergy. AI’s deep integration facilitates the emergence of a higher-order, AI-augmented ecologically coupled system. Within this system, AI transcends its traditional role as a passive tool, evolving into an agentive entity capable of actively generating, regulating, and catalyzing environmental affordances. Thus, this framework represents not a mere application of classical theory but a necessary evolution for the digital-intelligent age, specifically elucidating how AI reconfigures the structure, process, and intensity of multi-agent couplings.

To clarify its distinctiveness and to move beyond the limitations of existing constructs, ecological coupling is delineated from neighboring concepts (see Table 1). Unlike instructional alignment, which is mainly focusing on the pre-designed structural consistency, ecological coupling centers on the dynamic quality of interactive processes. Contrasted with social support, which is emphasizing resource provision, ecological coupling highlights the functional complementarity and synergy among AI, teachers, peers, and the environment. Differing from perceived AI integration which is targeting individual technology acceptance, ecological coupling examines the learner’s perception of the systemic collaborative efficacy between AI and the entire learning ecosystem.

**Table 1:** Comparative analysis of ecological coupling and related constructs.

Dimension	Ecological coupling (core construct)	Instructional alignment	Social support	Perceived integration of AI
Theoretical focus	Quality and dynamic process of interactions among multiple agents within a system	Consistency of predetermined structures among curricular elements	Acquisition of emotional, informational, or instrumental resources from others	Individual’s evaluation of a technology tool’s perceived usefulness and ease of use
Core relations	Multidirectional, dynamic, and co-constructive	Unidirectional, static, and predetermined	Primarily unidirectional or bidirectional	Unidirectional
Dynamic nature	High: Emphasizes real-time interaction, mutual adaptation, and co-evolution	Low: Focuses on the pre-implementation design state of instruction	Medium: Relies on a relatively stable network of support relations	Medium-low: Focuses on relatively stable attitudes and beliefs regarding use
Level of analysis	Mesosystem or Relational Network (focuses on interaction patterns among microsystems)	Curricular or Instructional System	Interpersonal or Group Level	Individual-Technology Interaction Level
Operationali-zation in this study	Measured via learners’ evaluations of relational quality concerning: AI-Teacher Synergy AI-Peer collaborative support AI-Environment affordance	Not addressed	Partially subsumed under “Peer Support,” but only as one facet within AI-Peer Collaborative support	Partially reflected by “Frequency of AI Use,” yet fails to capture the relational quality of the elements within the learning ecosystem
Key distinction	A multi-agent synergistic process mechanism that drives language learning engagement	A structural prerequis-ite for effective learning	A key resource that fosters language learning engagement	The psychological threshold of acceptance necessary for technology to exert its effects

In summary, we define the ecological coupling as: within the learning ecology, the emergent dynamic relational quality arising from the real-time interaction and functional complementarity among multiple learning elements such as AI, teachers, peers, and the environment. Its uniqueness lies in (1) transcending static, unidirectional logic to focus on multidirectional, sustained interaction, and (2) emphasizing synergistic emergence, where its core yield is the 1 + 1 > 2 synergistic affordance that provides additional impetus for learning.

### 1.3 Operationalization: interfaces and domains

To operationalize this construct, our framework distinguishes between two core components: the relational interfaces and the functional domains. Specifically, we examine three primary interfaces, namely AI-Teacher, AI-Peer, and AI-Environment. Correspondingly, the coupling manifests its effects across three domains. First, the cognitive domain (linked to AI-Teacher) focuses on how AI collaborates with teacher expertise to redistribute cognitive labor, optimizing conceptual development and higher-order thinking. Second, the social domain (linked to AI-Peer) concerns how AI mediates and catalyzes peer interactions, strengthening collaboration, dialogue, and community. Third, the situational domain (linked to AI-Environment) examines how AI integrates with the environment to co-construct an immersive, responsive learning context. Therefore, within this operational framework, high-quality ecological coupling is characterized by the successful integration of value across the cognitive, social, and situational domains, facilitated by productive coupling at the AI-Teacher, AI-Peer, and AI-Environment interfaces.

### 1.4 Research hypotheses and analytical strategy

Grounded in this integrated framework, we propose two hypotheses:

*H1*: The quality of ecological coupling between AI and other elements (teachers, peers, environment) mediates the relationship between AI use and language learning engagement.

*H2*: Distinct ecological configurations (typologies) can be identified from learners’ coupling experiences, and these configurations exhibit differential characteristics in both coupling patterns and engagement outcomes.

**Figure 1:**
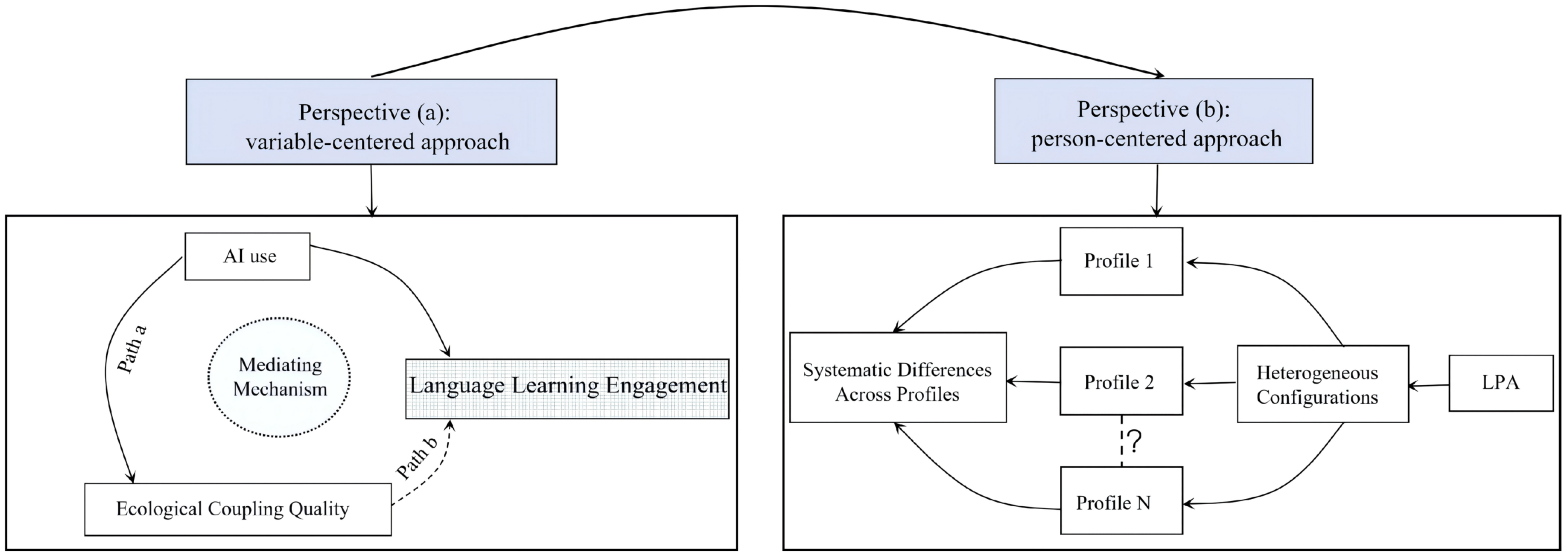
A conceptual framework for addressing the research questions.

To test these hypotheses, we employ a dual-perspective analytical strategy (see Figure 1):

- Perspective (a): A variable-centered approach using mediation analysis to quantitatively test the proposed overarching pathway (H1).
- Perspective (b): A person-centered approach using Latent Profile Analysis (LPA) to identify heterogeneous learner configurations and compare their differences (H2).

This integrated framework accounts for the overarching system structure, specific interaction mechanisms, and emergent heterogeneous patterns at the individual level. The dual-perspective strategy not only seeks to reveal the general mechanism of ecological empowerment but also to systematically investigate the diversity of learner experiences, offering a more comprehensive picture of AI’s complex role on language learning engagement.

## 2 Methods

Corresponding to the dual-perspective framework outlined in the introduction, this study employed an explanatory sequential mixed-methods design. The quantitative phase, involved a survey of 475 learners, simultaneously testing the variable-centered mediation model and performing the person-centered LPA to identify ecological profiles. The subsequent qualitative phase, based on interviews, provided depth and context to interpret the quantitative profiles and pathways.

### 2.1 Participants and procedure

This study was situated within the context of AI-enhanced informal language learning among the broad population of Chinese university students. To ensure that the participant sample was theoretically aligned with our research aims, which was investigating the ecological coupling between AI and the language learning ecosystem, a targeted sampling strategy was employed. We specifically focused on non-English major undergraduates for two primary reasons. First, they constituted the largest cohort of English learners in Chinese higher education, and their engagement with English was typically instrumental, for instance, for academic reading, test preparation, or future career needs, rather than literary or linguistic specialization. This instrumental orientation made them prime candidates for adopting AI tools as flexible, on-demand learning resources outside formal curricula. Second, their learning ecology was inherently multifaceted, characterized by the dynamic interplay of self-directed study, peer interaction, and teacher guidance in general English learning. This complex, real-world interactive context provided an ideal setting to critically examine how AI coupled with an existing learning system. Therefore, recruiting participants who were both active English learners and had prior experience with AI was essential to meaningfully address our core research questions.

Recruitment and sampling: Participants were recruited through convenience sampling. To facilitate access across multiple institutions, we collaborated with instructors teaching college English or related general courses at four comprehensive universities in East China. These instructors assisted in disseminating the study information and survey link to their students, ensuring potential participants received a clear explanation of the research aims, voluntary nature of participation, and their rights.

Inclusion criteria: To further operationalize the theoretical sample alignment, the following inclusion criteria were applied:

(1) Current enrollment as a non-English major undergraduate student actively engaged in English learning (e.g., through formal courses, self-study, or test preparation).
(2) Prior experience using AI tools specifically for English language learning (e.g., ChatGPT, Grammarly, AI tutors, or writing assistants) within the previous academic semester.
(3) Provision of informed consent for participation in the survey and optional follow-up interviews.

Exclusion criteria: The following exclusion criteria were applied to ensure data relevance and quality:

(1) Participants with no prior experience using AI for English learning.
(2) Participants who did not complete the survey in its entirety (e.g., submitted incomplete responses), as partial data would compromise the validity of the statistical models employed.
(3) Participants whose completion time fell below a reasonable threshold, indicating potential inattentive or insincere responding.
(4) Participants whose responses were contradictory before and after.

Procedure: The online survey was anonymous, administered via Wenjuanxing, a widely used and trusted online survey platform in China, ensuring anonymity and data security. A total of 482 non-English major undergraduates proceeded to the questionnaire. After applying the above exclusion criteria to the submitted responses, 475 valid participants (205 freshmen, 114 sophomores, 91 juniors, 65 seniors; 155 male, 320 female) were retained for the final analysis.

Upon completion, participants could proceed to a separate, unlinked form to volunteer for follow-up interviews. This form served a dual purpose: to register interest and to collect structured contextual data relevant to our research objectives. It included items on participants’ patterns of AI use such as frequency, AI apps and primary purposes, their initial perceptions of AI’s interaction with teachers, peers and the environment, and their self-assessment of AI’s impact on their English learning process, alongside basic demographics.

From this pool of volunteers, we employed a maximum variation purposeful sampling strategy. We analyzed the background information to identify individuals whose reported experiences promised to diverge meaningfully along the theoretical dimensions central to our study, specifically, the quality of AI-ecological coupling and the degree of felt empowerment. Eight participants (S1-S8) were selected to ensure our sample captured a broad spectrum of these pre-identified experiential patterns.

This strategy ensured that our qualitative sample was pre-structured for theoretical relevance and diversity even before the interviews began. Subsequently, the in-depth interviews confirmed and richly elaborated upon these initial patterns.

### 2.2 Justification for the qualitative sample size and selection

The selection of eight participants for the in-depth interviews was a deliberate decision grounded in the explanatory purpose of the sequential design and established qualitative research principles. Our primary strategy was maximum variation purposeful sampling. By analyzing the structured background information, we specifically selected individuals whose profiles promised maximum divergence along the core theoretical dimensions of AI-ecological coupling and perceived AI-empowerment. This ensured the sample was pre-structured for theoretical relevance and experiential diversity before the interviews began.

This strategically configured small sample of eight participants was determined to be both sufficient and optimal for our explanatory purpose. The decision was based on two key considerations inherent to qualitative inquiry. First, qualitative research prioritizes depth and contextual richness over statistical generalizability. This focused sample allowed for an extensive, detailed exploration of each participant’s lived experience, which is essential for mechanistic explanation. Second, and most critically, we achieved theoretical saturation within this sample. This was facilitated by our participant selection strategy. Following the initial survey, we analyzed a detailed information sheet completed by students to purposefully select eight interviewees who represented the widest possible range of experiences relevant to our core constructs (e.g., AI coupling and empowerment). This deliberate, criteria-driven approach ensured the sample was primed for achieving theoretical saturation efficiently. During data collection and analysis, we observed that after approximately seven interviews, no substantively new themes or theoretical insights were emerging regarding the core pathways linking AI use, ecological coupling, and engagement. The eighth interview served to confirm and consolidate the established framework. Consequently, additional interviews were deemed unnecessary. This sample was thus precisely suited to its task: to generate the rich, nuanced explanations required to interpret and elaborate upon the quantitative findings, thereby fulfilling the explanatory purpose of the sequential design and enhancing the credibility of the integrated study.

### 2.3 Research design and analytic strategy

The analysis proceeded sequentially. The quantitative phase operationalized the dual perspectives outlined in our conceptual framework. First, a variable-centered mediation analysis tested the hypothesized ecological pathway (H1). Second, a person-centered latent profile analysis (LPA) was conducted to identify distinct subgroups based on patterns of AI-ecological coupling (H2), followed by multivariate comparisons to validate profile differences. The subsequent qualitative phase provided contextual depth to interpret the quantitative findings.

### 2.4 Measures

#### 2.4.1 Operationalization of core constructs and scale development

The quantitative survey comprised several rigorously developed scales. Given the innovative nature of the core constructs, available validated scales were limited. Consequently, measurement tools were developed based on our theoretical framework (EST and EAT) through a three-phase procedure: (1) theoretical grounding and initial item generation, (2) an iterative expert review process incorporating student feedback, and (3) full-sample psychometric validation.

##### 2.4.1.1 Stage 1: theoretical grounding and initial item generation

Based on the theoretical frameworks of Ecological Systems Theory (EST) and Affordance Theory (EAT), we systematically reviewed domestic and international literature to clarify the connotations of AI empowerment, disempowerment risks, L2 learning engagement, and ecological coupling in the context of college English learning. We critically referenced existing validated scales, including Xu et al.’s (2025a, 2025b) scales for student–student, teacher-student, and human-AI interaction, as well as scales related to online learning engagement and technological affordance (Abbasi et al., 2024; Liu et al., 2024; Ma et al., 2022). Considering the characteristics of Chinese college students and the specific context of AI-assisted English learning, we initially formulated separate item pools for each scale: an 8-item pool for Perceived AI-Ecological Coupling Quality, an 8-item pool for AI-Empowerment Perception, and an 8-item pool for AI-Disempowerment Perception (24 items in total before refinement). Among these, the structure of the Coupling Quality scale was explicitly derived from our operational framework, with items designed to capture learners’ perceptions across the three relational interfaces (AI-Teacher, AI-Peer, AI-Environment) and their functional value in the corresponding domains (cognitive, social, situational).

##### 2.4.1.2 Stage 2: iterative expert review process

This phase was designed to maximize content validity and involved two key activities conducted in sequence.

Before formal expert review, we conducted informal discussions with a small group of students (*n* = 16) who matched our target population. The purpose was to identify any immediate issues with item comprehensibility and wording from a learner’s perspective. Based on their feedback, we refined the item pools to enhance clarity, which included the removal of one potentially ambiguous item (C7).

To ensure the scales’ theoretical alignment, cultural appropriateness, and content validity, we invited a panel of 5 experts (2 in Educational Psychology, 2 in Educational Technology, and 1 in Second Language Acquisition) to conduct two rounds of independent reviews. The experts evaluated each item based on three criteria: clarity of expression, consistency with EST and EAT frameworks, and cultural relevance to Chinese college students. Controversial items (C1 and C8) were thoroughly discussed: C1 was slightly revised to enhance clarity, and C8 was deleted for failing to meet theoretical alignment standards. This expert review process further calibrated the scales’ structure and content, reducing measurement error and improving construct validity. This combined process resulted in the final scales, comprising 22 items in total (a 6-item pool for Perceived AI-Ecological Coupling Quality Scale, an 8-item pool for AI-Empowerment Perception Scale, and an 8-item pool for AI-Disempowerment Perception Scale).

##### 2.4.1.3 Stage 3: full-sample psychometric validation

The final scales were administered in the main study (*N* = 475). As detailed in Sections 2.3.3–2.3.5, confirmatory factor analysis confirmed the excellent structural validity, and all reliability and validity indices (CR, AVE) met or exceeded established thresholds.

#### 2.4.2 AI use frequency

AI use frequency was assessed via a single-item measure, operationalized as the self-reported frequency of using AI tools for language learning. This operationalization was theoretically grounded from an ecological perspective, where frequent AI use was conceptualized not as a direct determinant of language learning engagement, but as a pragmatic initiating condition that activated the broader learning ecosystem. It signified the learner’s entry into and sustained immersion within an AI-augmented environment, thereby creating the necessary field for subsequent interactions with teachers, peers, and learning materials to unfold.

The adoption of a single-item measure was justified by the following interconnected considerations: theoretical alignment, empirical parsimony, and analytical focus. Theoretically, it corresponded to the role of a triggering mechanism within EST (Bronfenbrenner, 1979). The ecological impact of any element depended not merely on its presence but on its dynamic interaction with other system components. This ecological perspective aligned with Kurt Lewin’s (1936) concept of “life space,” which emphasized the continuous interplay between the individual and the environment, sustained activity was essential to transform a potential resource into functional momentum. Thus, adequate AI use frequency served as a prerequisite, enabling the coupling processes with teacher support, peer collaboration, and environmental affordances that collectively drove language learning engagement. Empirically, this approach maintained parsimony and aligned with the study’s core analytical focus. Given that the primary investigative target was the quality of coupling mechanisms rather than the granular details of AI usage patterns such as specific durations or functional subtypes, a concise measure avoided unnecessary complexity while sufficiently capturing the critical construct of ecological activation. The functional validity of this measure was supported by the robustness of the subsequent analytical findings, as presented in the results section.

#### 2.4.3 Perceived AI-ecological coupling quality scale

This hypothesized mediator captured learners’ subjective assessment of how effectively AI interacts with and aligns key ecological components. It was measured using the Perceived AI-Ecological Coupling Quality Scale (6 items; C1-C6), which assessed multi-agent interaction for AI-ecological coupling. Specifically, it assessed the perceived coupling quality across three relational interfaces: AI-Teacher interface (C1, C2, focusing on cognitive synergy and labor redistribution), AI-Peer interface (C3, C4, focusing on social mediation and collaboration), and AI-Environment interface (C5, C6, focusing on contextual immersion and responsiveness). The scale exhibited excellent internal consistency (Cronbach’s *α* = 0.954). Confirmatory factor analysis supported its strong structural validity, with all fit indices meeting recommended thresholds (*χ*^2^/df = 2.751, CFI = 0.997, TLI = 0.991, RMSEA = 0.061, SRMR = 0.0236). All standardized factor loadings ranged from 0.878 to 0.938. Both composite reliability (CR = 0.92–0.95) and average variance extracted (AVE = 0.78–0.83) substantially exceeded their respective critical thresholds of 0.7 and 0.5, collectively demonstrating high convergent validity and internal consistency reliability. These results confirm the scale’s robust psychometric properties.

#### 2.4.4 AI-empowerment perception scale

The dependent variable, learning engagement in the context of human-AI collaboration, was operationalized as AI-Empowerment Perception and measured using an 8-item scale (E1-E8). Its items corresponded to the four engagement dimensions (behavioral, cognitive, emotional, social). The scale demonstrated excellent model fit (*χ*^2^/df = 1.98, CFI = 0.996, TLI = 0.993, RMSEA = 0.045, SRMR = 0.007), with all factor loadings being significant and substantial (ranging from 0.829 to 0.939). The scale exhibited strong psychometric properties, with composite reliability values ranging from 0.833 to 0.918 and average variance extracted (AVE) estimates ranging from 0.714 to 0.848, supporting its reliability and convergent validity. The scale demonstrated excellent internal consistency (Cronbach’s *α* = 0.957).

#### 2.4.5 AI-disempowerment perception scale

The AI-Disempowerment Perception Scale was developed to assess multidimensional risks associated with AI-driven disempowerment across four dimensions: cognitive, behavioral, emotional, and social risks. CFA demonstrated that the theoretically grounded four-factor model provided a good fit to the data, as evidenced by the following indices: CFI = 0.988, TLI = 0.977, RMSEA = 0.073, SRMR = 0.020. The ratio of chi-square to degrees of freedom (*χ*^2^/df = 3.50) was slightly elevated, which was a known phenomenon in large samples where even trivial discrepancies became statistically significant; however, the robustness of the other fit indices supported the acceptability of the model fit (Kline, 2016). All items loaded strongly on their respective factors (standardized loadings > 0.80). Furthermore, the scale exhibited high internal consistency (Cronbach’s *α* = 0.926), high composite reliability (CR = 0.819–0.898), and sound convergent validity (AVE = 0.689–0.816). These results collectively confirmed that the scale possesses robust reliability and validity for use in subsequent research.

#### 2.4.6 Qualitative interview protocol

Semi-structured interviews were conducted with selected participants to elicit rich, narrative data. The protocol was designed to explore learners’ lived experiences of AI-mediated learning, focusing on their perceptions of interactions with teachers and peers, the alignment of AI with the learning environment, and specific instances of feeling empowered or disempowered.

### 2.5 Data analysis

#### 2.5.1 Preliminary and mediation analysis (testing H1)

Data analysis commenced with preliminary analyses (descriptive statistics, correlations) in SPSS 26.0. To test H1, a simple mediation analysis was conducted using Model 4 of the SPSS PROCESS macro with 5,000 bootstrap samples. The model specified AI use frequency as the independent variable, the mean score of the items of Perceived Coupling Quality Scale as the mediator, and the mean score of the items of AI-Empowerment Perception Scale as the dependent variable. The use of global composite scores was theoretically and methodologically justified. First, this approach directly aligned with the holistic nature of H1, which examined relationships between overarching constructs rather than their sub-dimensions. Second, it maintained statistical parsimony and power, avoiding the complexity and multicollinearity risks associated with modeling all sub-dimensions separately. Finally, the strong psychometric properties of the scales, specifically, their validated multidimensional structure and high internal consistency (Cronbach’s *α* = 0.954 for coupling quality; 0.957 for engagement), supported the aggregation of sub-dimensions into reliable higher-order composites.

#### 2.5.2 Latent profile and follow-up analyses (testing H2)

To test H2 and address the limitation of assuming population homogeneity in the mediation model, we employed a person-centered approach, Latent Profile Analysis (LPA) in Mplus 8.3. The six items of the Perceived AI-Ecological Coupling Quality Scale (C1-C6), which operationalize the three relational interfaces, namely AI-Teacher, AI-Peer, AI-Environment, served as continuous indicators to identify subgroups based on distinctive coupling patterns.

To determine the optimal number of latent profiles, we systematically estimated and compared 2-, 3-, and 4-profile solutions. Model estimation employed 1,000 random starts and 250 final-stage optimizations to ensure the robustness of the solutions and avoid convergence on local maxima. Model selection was guided by a comprehensive evaluation of statistical fit indices (including AIC, BIC, aBIC, and LMR test), entropy (as a measure of classification accuracy), and the substantive interpretability and theoretical utility of the profiles. Following established guidelines, solutions with identification issues or containing profiles with an excessively small sample size (e.g., < 5%) were deemed untenable.

To validate and characterize the emergent profiles, we planned a two-step procedure. First, a Multivariate Analysis of Variance (MANOVA) was conducted to examine whether the profiles exhibited systematic differences across a broader set of variables: the three dimensions of Perceived AI-Ecological Coupling Quality Scale, the four dimensions of AI-Empowerment Perception Scale, and the mean of the items of AI-Disempowerment Perception Scale. Profile membership served as the independent variable. Second, if the multivariate effect was significant, we planned to follow it with univariate ANOVAs and Games-Howell *post hoc* comparisons to pinpoint the specific differences between profiles on each variable.

#### 2.5.3 Qualitative data analysis

The interview data were analyzed using content analysis, and any coding discrepancies among the three researchers were resolved through discussion until consensus was achieved.

## 3 Results

### 3.1 Descriptive statistics and preliminary analysis

Prior to testing the main hypotheses, descriptive statistics and bivariate correlations for all study variables were examined (see Table 2). The mean scores for the core positive constructs were above the scale midpoint of 3: Perceived AI-Ecological Coupling Quality Scale (*M* = 3.68, SD = 0.73) and AI-Empowerment Perception Scale (*M* = 3.61, SD = 0.76). The mean for AI-Disempowerment Perception Scale fell below the scale midpoint (*M* = 2.75, SD = 0.76). All variables demonstrated sufficient variability for subsequent analyses. As was shown in Table 2, the absolute values of skewness (range = −0.52 to 0.11) and kurtosis (range = −0.94 to 1.35) for all variables were well within the conventional thresholds for assuming normality (i.e., |skewness| < 3 and |kurtosis| < 10; Kline, 2015), indicating no severe deviations from a normal distribution.

**Table 2:** Descriptive statistics, normality indices, and correlations for key variables.

Variable	*M*	SD	Skewness	Kurtosis	1	2	3	4	5	6	7	8	9	10	11
1. AI usage frequency	3.17	1.27	−0.10	−0.94	(–)										
2. Perceived AI-ecological coupling quality (overall)	3.68	0.73	−0.41	1.28	0.15^**^	**(0.954)**									
3. AI-empowered perception (overall)	3.61	0.76	−0.48	1.35	0.22^**^	0.67^**^	**(0.957)**								
4. AI-disempowerment perception (overall)	2.75	0.76	0.11	0.65	0.01	−0.04	−0.01	**(0.926)**							
Coupling quality dimensions															
5. AI-Teacher	3.72	0.78	−0.40	0.84	0.11^*^	0.94^**^	0.58^**^	−0.00	**(0.903)**						
6. AI-Peer	3.69	0.80	−0.36	0.64	0.15^**^	0.95^**^	0.65^**^	−0.02	0.84^**^	**(0.932)**					
7. AI-Environment	3.62	0.75	−0.31	0.92	0.14^**^	0.93^**^	0.67^**^	−0.09	0.79^**^	0.84^**^	**(0.908)**				
Learning engagement dimensions															
8. Behavioral	3.65	0.79	−0.49	1.11	0.21^**^	0.64^**^	0.91^**^	0.00	0.56^**^	0.63^**^	0.64^**^	**(0.833)**			
9. Cognitive	3.60	0.82	−0.50	0.87	0.22^**^	0.62^**^	0.93^**^	−0.03	0.53^**^	0.60^**^	0.62^**^	0.84^**^	**(0.882)**		
10. Emotional	3.59	0.84	−0.43	0.71	0.18^**^	0.60^**^	0.93^**^	−0.00	0.53^**^	0.57^**^	0.60^**^	0.78^**^	0.82^**^	**(0.917)**	
11. Social	3.59	0.82	−0.52	1.08	0.22^**^	0.63^**^	0.93^**^	−0.01	0.53^**^	0.61^**^	0.63^**^	0.77^**^	0.80^**^	0.86^**^	**(0.907)**

*N* = 475. *M*, Mean; SD, Standard Deviation. Reliability coefficients (Cronbach’s Alpha) are presented in bold parentheses along the diagonal. AI Usage Frequency was a single-item measure, hence no reliability coefficient is provided. All study variables demonstrated acceptable univariate normality, with absolute values of skewness < 3 and kurtosis < 10. ^*^p < 0.05. ^**^p < 0.01.

The correlation matrix revealed a pattern consistent with the theoretical framework. AI use frequency was positively correlated with both overall perceived AI-ecological coupling quality (*r* = 0.15, *p* < 0.01) and overall AI-empowered perception (*r* = 0.22, *p* < 0.01). A strong positive correlation was observed between overall perceived AI-ecological coupling quality and overall AI-empowered perception (*r* = 0.67, *p* < 0.01). Furthermore, the overall scores for both AI-empowered perception and coupling quality were highly correlated with their respective dimensional scores (all r ≥ 0.91 and all r ≥ 0.93, respectively), supporting the internal coherence of these composite constructs. The strong intercorrelations among dimensions within each construct also indicated conceptual relatedness. As anticipated, AI-disempowerment perception were not significantly correlated with AI use frequency, coupling quality, or AI-empowered perception, confirming its distinctiveness within the model.

### 3.2 Mediation analysis (H1)

#### 3.2.1 Quantitative findings

Mediation analysis confirmed H1. Perceived AI-ecological coupling quality partially mediated the positive link between AI use frequency and AI-empowerment perception (see Table 3). Total effect (c) = 0.134, 95% CI [0.081, 0.187]; direct effect (c′) = 0.078, 95% CI [0.038, 0.118]; indirect effect = 0.056, 95% Bootstrap corrected and accelerated (BCa) CI [0.019, 0.096]. The standardized indirect effect was 0.092, implying a 1-SD rise in AI use frequency yielded a 0.092-SD gain in AI-empowerment perception via perceived AI-ecological coupling quality, accounting for 41.8% of the total effect, suggesting a moderate degree of mediation (following common interpretation guidelines for proportion mediated). Path a (AI use frequency→perceived AI-ecological coupling quality) was statistically significant but small (*β* = 0.150, *p* < 0.01, *f*^2^ = 0.023), whereas Path b (perceived AI-ecological coupling quality → AI-empowerment perception) was statistically significant, with a larger effect size (*β* = 0.670, *p* < 0.001, *f*^2^ = 0.501).

**Table 3:** Results of the mediation analysis (*N* = 475).

Effect/path	Unstandardized effect (SE)	Bootstrap 95% CI	Standardized effect (*β*)	Effect size metrics	Interpretation
Total effect (c)	0.134 (0.027)	[0.081, 0.187]	0.220	–	–
Direct effect (c′)	0.078 (0.020)	[0.038, 0.118]	0.128	–	–
Indirect effect (a × b)	0.056 (0.020)	[0.019, 0.096]	0.092	Proportion Mediated: 41.79%	Moderate
Path a (X → M)	0.083 (0.026)	[0.032, 0.135]	0.150	Cohen’s *f*^2^ = 0.023	Small
Path b (M → Y)	0.676 (0.035)	[0.606, 0.745]	0.670	Cohen’s *f*^2^ = 0.501	Large

SE, standard error; CI, confidence interval. The indirect effect and its CI are derived from bootstrap sampling with 5,000 repetitions (Efron and Tibshirani, 1993). All values are reported to three decimal places. X = AI Use Frequency; M = Perceived AI-Ecological Coupling Quality; Y = AI-Empowerment Perception (Language Learning Engagement). Effect size (Cohen’s *f*^2^) interpretations follow Cohen’s (1988) guidelines: small (<0.02), medium (≥0.02 and <0.15), and large (≥0.15).

To provide contextualized illustration for the quantitative pathways identified above, student interview data were analyzed.

##### 3.2.1.1 Direct pathway

Interview narratives provided concrete examples of how AI use frequency linked to engagement. One participant described a cycle of using AI, consulting the teacher, and comparing outputs, which sustained their writing practice: “When using AI to learn English writing, I encountered the issue of receiving overly general feedback. I continued practicing sought help from my English teacher compared it with the model essays generated by AI. Gradually, my English writing skills were improved” (S1).

##### 3.2.1.2 Mediating pathway

Participants described perceiving AI as part of a multi-agent system. For example, one student stated: “AI served as an aid, the teacher provided guidance, and classmates were the main actors in mutual communication” (S7). Another similarly described: “AI provided the framework, the teacher provided guidance, and classmates exchanged ideas” (S4).

Narratives further depicted students proactively refining their interaction with AI to improve its functional integration. One student detailed adjusting AI’s output to match their proficiency level: “In reciting and writing, I found that the vocabulary provided by AI exceeded my acceptance. After several adjustments, I regulated the vocabulary within the range of CET-4 and CET-6” (S5). Another student described strategically redefining AI’s role: “I successfully transformed AI from a tolerant chat partner into a precise error-correction tool, effectively improving my learning efficiency.” (S8).

### 3.3 Latent profile analysis (LPA): emergent ecological configurations (H2)

To examine potential heterogeneity in students’ experiences beyond the averaged mediation pattern, a Latent Profile Analysis (LPA) was conducted. The analysis was grounded in the operational framework of ecological coupling, utilizing the perceived quality of coupling at the three key relational interfaces, namely AI-Teacher, AI-Peer, and AI-Environment, as profile indicators.

#### 3.3.1 Identification, validation, and characterization of profiles

**Figure 2:**
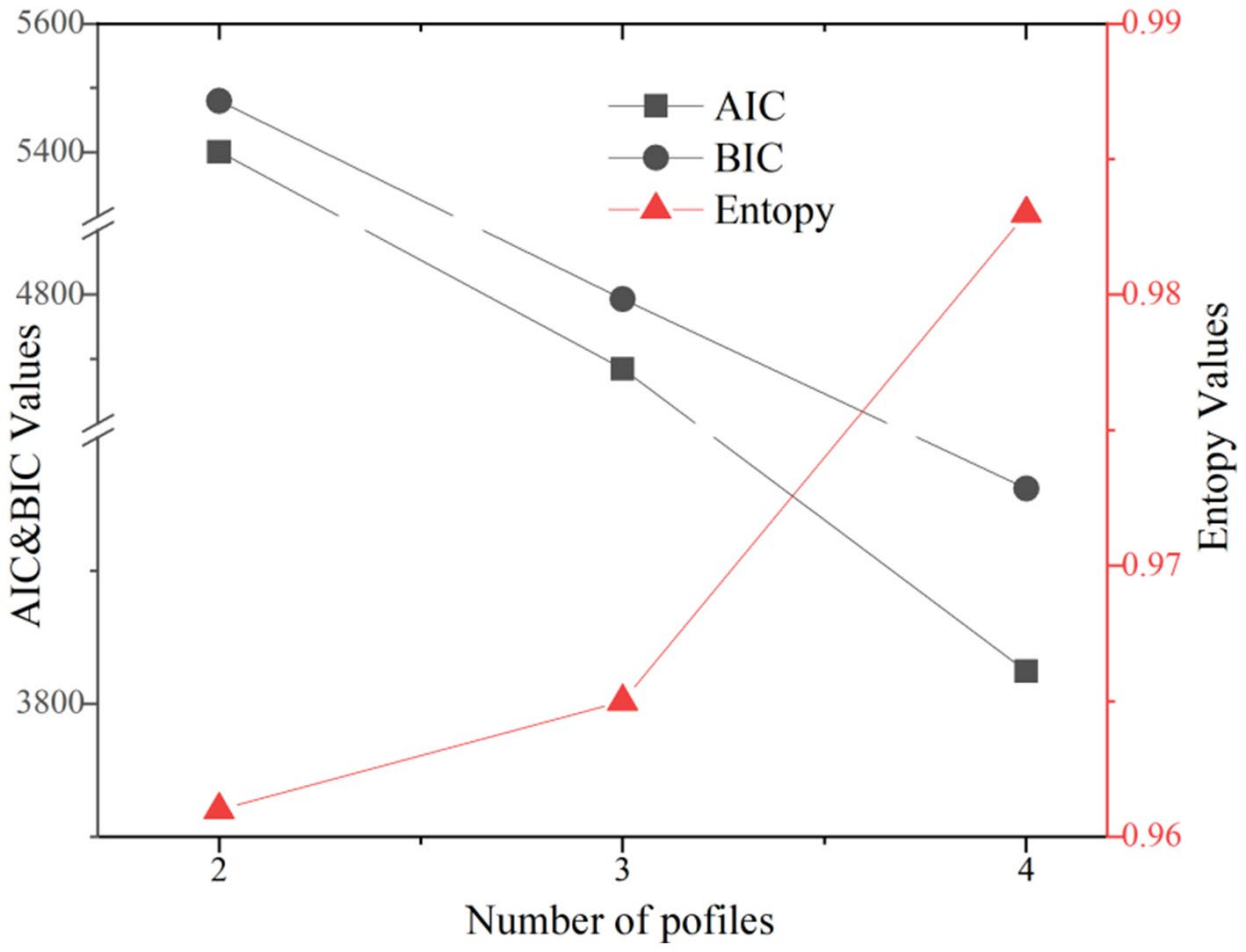
Latent profile model selection criteria.

**Figure 3:**
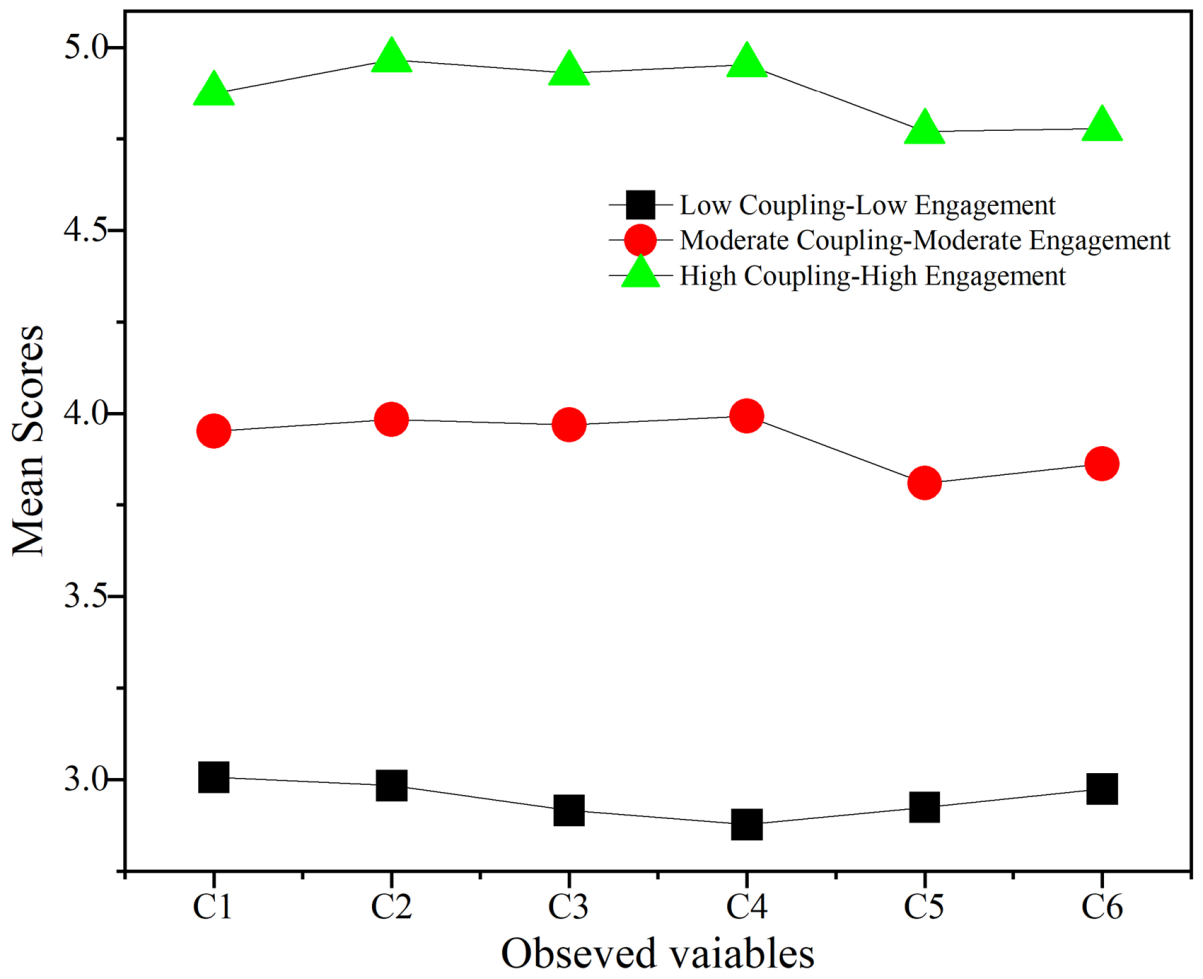
Observed variable mean profiles for the three-profile model.

A three-profile model was identified as optimal (see Table 4; Figures 2 and 3). The selected model showed excellent classification accuracy (entropy = 0.965) and significant improvement over a two-profile solution. Although a four-profile model showed marginally better AIC and BIC, it was rejected due to identification issues and an unacceptably small profile (*n* = 11, 2.32%). Guided by established methodological conventions for latent profile analysis, which advocate for a descriptive and data-driven nomenclature (Xu and Yang, 2024; Li et al., 2022; Marsh et al., 2009), the final three-profile solution was labeled strictly according to the two key dimensions that defined the groups: ecological coupling quality and language learning engagement. The final profiles were labeled as: Profile 1: Low Coupling-Low Engagement (*n* = 183, 38.7%), Profile 2: Moderate Coupling-Moderate Engagement (*n* = 228, 47.7%), and Profile 3: High Coupling-High Engagement (*n* = 64, 13.6%) (see Table 5).

**Table 4:** Comparison of latent profile model fit indices.

Fit indices	2-profile model	3-profile model	4-profile model
AIC	5,400.455	**4,684.784**	3,824.238
BIC	5,479.558	**4,793.030**	3,961.627
Sample-size adjusted BIC	5,419.255	**4,710.510**	3,856.890
Entropy	0.961	**0.965**	0.983
VLMR test *p*-value	0.0002	0.1800	0.2178
LMR test p-value	0.0002	0.1851	0.2234
Smallest profile proportion	41.09%	13.59%	2.32%
Model issues	None	None	Present

Bold values indicate optimal indices for the recommended model.

**Table 5:** Parameter estimates for the 3-profile model.

Variable	Profile 1 (low-level)	Profile 2 (medium-level)	Profile 3 (high-level)
C1	3.007	3.952	4.875
C2	2.984	3.984	4.967
C3	2.916	3.970	4.931
C4	2.878	3.994	4.954
C5	2.925	3.810	4.770
C6	2.975	3.864	4.779
Profile proportion	38.7%	47.7%	13.6%
Actual *N*	183	228	64

All parameter estimates are statistically significant (*p* < 0.001). Profile proportions are based on estimated posterior probabilities. Model fit indices support the 3-profile model (AIC = 4684.784, BIC = 4793.030, Entropy = 0.965).

The distinctiveness of the three profiles was rigorously validated. A MANOVA with profile membership as the independent variable and the eight key outcome variables as dependents revealed a significant multivariate effect (Wilks’ *Λ* = 0.160, *F*(16, 930) = 87.18, *p* < 0.001, partial *η*^2^ = 0.600) (see Table 6). Subsequent univariate ANOVAs confirmed that all eight variables differed significantly across all profiles (all *p* < 0.001). Games-Howell *post hoc* comparisons established a consistent ordinal relationship for every variable: Profile 1 < Profile 2 < Profile 3 (see Table 7).

**Table 6:** Multivariate analysis of variance (MANOVA) for ecological coupling and learning outcome variables by latent profile membership.

Effect	Statistic	Value	*F*	Hypothesis df	Error df	*p*	partial *η*^2^
Intercept	Pillai’s trace	0.992	7,248.41	8	465	<0.001	0.992
	Wilks’ lambda	0.008	7,248.41	8	465	<0.001	0.992
Latent profile	Pillai’s trace	**0.864**	**44.29**	**16**	**932**	**<0.001**	**0.432**
	Wilks’ lambda	**0.160**	**87.18**	**16**	**930**	**<0.001**	**0.600**
	Hotelling’s trace	**5.101**	**147.93**	**16**	**928**	**<0.001**	**0.718**
	Roy’s largest root	**5.071**	**295.41**	**8**	**466**	**<0.001**	**0.835**

The independent variable was latent profile membership. The dependent variables were AI-teacher coupling, AI-peer coupling, AI-environment coupling, behavioral engagement, cognitive engagement, emotional engagement, social engagement, and disempowerment risks. All multivariate tests indicated a significant main effect of profile membership, *p* < 0.001.

**Table 7:** Differences in ecological coupling, learning engagement, and disempowerment risks across ecological profiles.

Variable	Profile 1 (*n* = 183)	Profile 2 (*n* = 228)	Profile 3 (*n* = 64)	*F*(2, 472)	*p*	Partial *η*^2^	Games-Howell *Post Hoc*
Ecological coupling quality							
AI-teacher	2.997 (0.5559)	3.965 (0.3494)	4.922 (0.2705)	537.430	<0.001	0.695	1 < 2 < 3
AI-peer	2.885 (0.4723)	3.989 (0.2833)	4.945 (0.1808)	929.529	<0.001	0.798	1 < 2 < 3
AI-environment	2.954 (0.5128)	3.829 (0.3803)	4.789 (0.3652)	473.404	<0.001	0.667	1 < 2 < 3
Learning engagement							
Behavioral	3.164 (0.7247)	3.816 (0.5443)	4.430 (0.8585)	99.693	<0.001	0.297	1 < 2 < 3
Cognitive	3.098 (0.7458)	3.805 (0.5575)	4.313 (0.9365)	91.581	<0.001	0.280	1 < 2 < 3
Emotional	3.126 (0.7545)	3.763 (0.6408)	4.328 (0.9268)	76.324	<0.001	0.244	1 < 2 < 3
Social	3.104 (0.7169)	3.772 (0.6034)	4.352 (0.9330)	89.817	<0.001	0.276	1 < 2 < 3
Disempower-ment Risks	2.8108 (0.60813)	2.7626 (0.73220)	2.5566 (1.14563)	2.693	0.069	0.011	n.s.

Values in parentheses are standard deviations. All post-hoc comparisons are significant at *p* < 0.05. n.s., not significant.

Based on the established quantitative patterns, the three distinct profiles were interpreted and named as follows (see Table 8). The following section will elaborate on these profiles qualitatively.

**Table 8:** Summary and characterization of the three ecological profiles.

Profile name and size	Defining quantitative signature	Qualitative illustration and behavioral manifestation	Theoretical interpretation
Profile 1: Low Coupling-Low Engagement profile (*n* = 183, 38.7%)	Lowest scores on all AI-ecological coupling dimensions (AI-Teacher, AI-Peer, AI-Environment). Lowest levels across all four dimensions language learning engagement. AI-Disempowerment risks comparable to other profiles (n.s.).	1. Frustration and Disconnection: “…Uninstalled the AI software.” (S2) 2. Perceived Deskilling: “After using the sentences generated by AI… I found myself unable to write them on my own, so I stopped using AI later.” (S3) 3. anifestation: Behavioral disengagement and AI abandonment.	A breakdown in the perception-interpretation-action cycle (Van Lier, 2004). Weak AI-ecological coupling fails to activate empowering affordances, leading to a state of disengagement and active disempowerment.
Profile 2: Moderate Coupling-Moderate Engagement profile (*n* = 228, 47.7%)	Moderate-to-High scores on all AI-ecological coupling dimensions. Robust learning engagement across all four dimensions, significantly higher than Profile 1 (*p* < 0.001).	Functional Orchestration: “AI served as an aid, the teacher provided guidance, and classmates were the main actors in mutual communication….” (S7) Sequential Integration: “AI completed the first draft, I revised it myself, the teacher reviews it, and classmates helped with proofreading.” (S6) Manifestation: Effective use of AI within established learning structures and works.	Exemplifies the actualization of ecological affordances (Gibson, 1979) through functional integration. Represents a well-coupled ecosystem where technology complements human-centric processes. Underpins the profile’s robust and empowered engagement.
Profile 3: High Coupling-High Engagement profile (*n* = 64, 13.6%)	Highest scores on all dimensions of AI-ecological coupling (*p* < 0.001). Highest levels of empowered engagement across all four dimensions (*p* < 0.001). Lowest disempowerment risks (n.s.).	Strategic Redefinition: “I successfully transformed AI from a “tolerant chat partner” into a precise error-correction tool…”(S8) Synergistic Vision: “AI will become our exclusive immersive scenario coach and cross-cultural communication co-pilot….” (S1) Manifestation: Metacognitive oversight and proactive (re)design of the learning ecosystem around AI.	Exemplifies optimal complementarity (Gibson, 1979). Learners act as architects of their learning ecology, achieving AI-empowerment through superior ecological coupling and strategic resource orchestration.

The profile names and interpretations are grounded in the integration of quantitative patterns (see Table 7 for ANOVA details) and qualitative evidence. n.s., not significant; *p* > 0.05.

##### 3.3.1.1 Profile 1: low coupling-low engagement profile (*n* = 183, 38.7%)

This profile showed the lowest scores across all AI-ecological coupling and learning engagement dimensions. Quantitatively, it had the lowest mean scores on all three AI interface measures (AI-Teacher: *M* = 2.99, SD = 0.56; AI-Peer: *M* = 2.89, SD = 0.47; AI-Environment: *M* = 2.95, SD = 0.51). Its mean scores on the four AI-empowered language learning engagement dimensions (behavioral: *M* = 3.164, SD = 0.725; cognitive: *M* = 3.098, SD = 0.746; emotional: *M* = 3.126, SD = 0.755; social: *M* = 3.104, SD = 0.717) were also the lowest among the three profiles, with all pairwise comparisons to Profiles 2 and 3 being statistically significant (all *p* < 0.001).

Interview data from participants in this profile (S2, S3) included statements such as “Uninstalled the AI software” (S2) and “After using the sentences generated by AI for my composition, I found myself unable to write them on my own, so I stopped using AI later” (S3).

##### 3.3.1.2 Profile 2: moderate coupling-moderate engagement profile (*n* = 228, 47.7%)

This was the largest profile. It exhibited moderate-to-high mean scores on the AI interface measures (AI-Teacher: *M* = 3.97, SD = 0.35; AI-Peer: *M* = 3.99, SD = 0.28; AI-Environment: *M* = 3.83, SD = 0.38). The AI-empowered language learning engagement scores across all dimensions were also moderate-to-high (behavioral: *M* = 3.82, SD = 0.54; cognitive: *M* = 3.81, SD = 0.56; emotional: *M* = 3.76, SD = 0.64; and social: *M* = 3.77, SD = 0.60).

Interview data from participants in this profile (S6, S7) described AI use in specific workflows. For example, S6 outlined: “AI completed the first draft, I revised it myself, the teacher reviewed it, and classmates helped with proofreading.” S7 characterized the system as: “AI served as an aid, the teacher provided guidance, and classmates were the main actors in mutual communication.”

##### 3.3.1.3 Profile 3: high coupling-high engagement profile (*n* = 64, 13.6%)

This profile, the smallest in size, demonstrated the highest levels of AI-ecological integration. Learners in this profile reported the highest perceived coupling across all three interfaces (AI-Teacher: *M* = 4.92, SD = 0.27; AI-Peer: *M* = 4.95, SD = 0.18; AI-Environment: *M* = 4.79, SD = 0.37). Correspondingly, they also exhibited the highest scores across all four dimensions of AI-empowered language learning engagement (behavioral: *M* = 4.43, SD = 0.86; cognitive: *M* = 4.31, SD = 0.94; emotional: *M* = 4.33, SD = 0.93; social: *M* = 4.35, SD = 0.93).

Interview data from participants in this profile (S1, S8) included statements such as: “I successfully transformed AI from a tolerant chat partner into a precise error-correction tool. while also consulting grammar books to investigate the reasons” (S8), and “In the future, AI will become our exclusive immersive scenario coach and cross-cultural communication co-pilot, providing personalized training that is hard for humans to replicate” (S1).

## 4 Discussion

This study set out to unravel how AI empowers language learning engagement. Moving beyond a focus on mere usage frequency, we adopted an ecological lens to theorize AI as an interactive agent within the learning ecosystem. Our integrated findings, combining variable-centered and person-centered analyses, converge to support an AI-empowerment Configural Model. This model explains empowerment not as a direct outcome of technology use, but as an emergent property of the quality of AI’s ecological coupling with teachers, peers, and the environment.

The mediation analysis established the pivotal mechanistic role of this coupling. It confirmed that the perceived quality of AI-ecological coupling partially mediated the relationship between AI use frequency and engagement. While usage directly contributed, a substantial portion (41.79%) of its total effect operated through this qualitative relational pathway. This underscores that what matters is not just if AI is used, but how it is contextually integrated.

LPA then systematized the heterogeneous outcomes implied by this mechanism, moving from a general pathway to specific learner typologies. We identified three distinct, theoretically coherent profiles: Low Coupling-Low Engagement, Moderate Coupling-Moderate Engagement, and High Coupling-High Engagement. These profiles do not merely represent different quantities of engagement; they embody qualitatively different stages of ecosystem integration. They form a progression defined by shifts in how learners configure their relationships with AI and other ecological elements.

In conclusion, the variable-centered result and the person-centered result are two sides of the same theoretical coin. Together, they validate the core proposition of our AI-empowerment Configural Model: what empowered engagement is co-produced through specific, qualitatively distinct configurations of AI within the learning ecology. The following sections elaborate on the nature of these configurations, their theoretical implications, and the model’s utility for research and practice.

### 4.1 The centrality of ecological coupling: beyond use frequency

A central finding of this study is the robust mediating role of perceived AI-ecological coupling quality. The relationship between AI use frequency and learning engagement is partially carried through this mediating mechanism, as evidenced by a significant indirect effect (0.056, 95% BCa CI [0.019, 0.096]) alongside a persistent direct effect (0.078). The proportion mediated is 41.79%—a moderate-to-large effect (Preacher and Kelley, 2011), meaning nearly 42% of AI’s total influence on learning engagement is channeled through learners’ perception of coupling quality. This highlights that the quality of AI’s integration with teachers, peers and environment is at least as critical as the use frequency, reframing the key driver of engagement from mere AI adoption to the quality of ecological coupling.

Path-specific effect sizes further reinforce this conclusion. The effect of coupling quality on learning engagement (Path b) is large (Cohen’s *f*^2^ = 0.501), far exceeding conventional thresholds for a large effect (Cohen, 1988), whereas the effect of AI use frequency on coupling quality (Path a) is small (*f*^2^ = 0.023). The stark contrast between these two paths not only shifts the focus toward technology-in-context but also substantiates the core tenets of EST, which posits that developmental outcomes arise from synergistic interactions among ecosystem elements rather than from isolated factors.

Crucially, this pattern of results validates our methodological approach. Despite the parsimonious measurement of AI use frequency, the data analysis clearly revealed the overwhelming importance of coupling quality. This indicates that the effect of the ecological coupling is robust enough that even a simplified measure of the initiating condition cannot obscure its status as the key mechanism. This finding empirically corroborates the validity of operationalizing frequency as a proxy for ecological activation in this study.

These results align with and quantitatively extend the ecological perspective advanced by scholars such as Jiang (2025). By moving beyond enumerating influencing factors to establishing an empirical hierarchy among them, this study offers a more nuanced account of the heterogeneous outcomes of AI integration reported in the literature (e.g., Huang et al., 2023; Karataş et al., 2024), suggesting that variation in coupling quality may be a key source of such divergence.

The path contrast also reframes the primary challenge of AI-enabled learning: the barrier is not tool adoption per se, but the subsequent achievement of meaningful ecological integration. This insight shifts the theoretical emphasis from the initial use of AI tools to the strategic cultivation of high-quality coupling, with clear implications for the focus of pedagogical support.

Ultimately, these findings compel a reconceptualization of AI from a tool to be used into a relational component that must be woven into the learning ecology. This reconceptualization, centered on coupling quality, is formally systematized in our AI-empowerment Configural Model (see Section 4.3), which explicates how this core mechanism explains heterogeneous outcomes.

### 4.2 A typology of AI-ecological engagement: from ecological disconnection to agentic synergy

Moving beyond a variable-centered perspective, our LPA identified three distinct learner profiles, namely Low Coupling-Low Engagement, Moderate Coupling-Moderate Engagement, and High Coupling-High Engagement, systematize the heterogeneous effects of AI documented in the literature (e.g., Huang et al., 2023; Karataş et al., 2024). These profiles represent conceptually discrete types, yet they form a theoretically meaningful sequence reflecting progressively advanced stages of integration within the AI-ecosystem. This progression is defined not merely by increasing engagement levels, but by qualitative shifts in how learners configure their relationships with AI, teachers, peers, and the learning environment.

The three latent profiles form a qualitative continuum, from structural disconnection to strategic orchestration, that moves beyond the variable-centered mediation model. This progression is defined not by the frequency of AI use but by a fundamental shift in the core mechanism itself: from passive or failed coupling to an agentically constructed, high-quality ecological coupling.

At one pole, the Low Coupling-Low Engagement profile illustrates a critical dysfunction in the learning ecosystem, manifesting in dual pathways of disempowerment. First, it exemplifies a breakdown in the perception-action cycle, where the weakest perceived coupling triggers a cascading disengagement effect. This is characterized not by passive non-use but by active abandonment, driven by a critical mismatch between expectation and reality (e.g., frustration that “AI lacked its own ideas,” S3). Such disappointment leads to decisive discontinuation (“Uninstalled the AI software,” S2), which forecloses any opportunity to discover AI’s potential value. Second, even in instances of continued use, a state of profound ecological decoupling prevails. Users within this profile report the lowest levels of teacher guidance, peer collaboration, and environmental support among all groups. This misalignment results in the least empowered form of engagement across all dimensions. Collectively, these manifestations substantiate the disempowerment narrative advanced in prior literature (Karatas et al., 2024; Jiang et al., 2022), crucially locating its origin in failed ecological coupling rather than in deficiencies of the AI tool itself. The profile demonstrates that in the absence of supportive coupling, AI not only fails to empower but can inadvertently exacerbate educational isolation and ineffectiveness, confining learners to a state of passive participation and validating the theoretical risks associated with poorly integrated learning technologies.

At the opposite pole of this spectrum, the High Coupling–High Engagement profile embodies an effective form of human-AI symbiosis. As the smallest subgroup (*n* = 64, 13.6%), it is defined by a configuration featuring the most frequent AI use and the highest perceived ecological coupling, a pattern that corresponds to the most elevated empowerment scores across all profiles. Within this configuration, AI functions as a cognitive mindtool (Jonassen, 1996), offloading routine tasks to free cognitive and social resources for deeper engagement. Learners actively reconfigure AI’s role to serve specific learning objectives. This is illustrated by one participant who “transformed AI from a tolerant chat partner into a precise error-correction tool” (S8) while deliberately coupling it with other resources such as grammar books. Such practices reflect an advanced mental model of AI as an active collaborator (e.g., a “co-pilot,” S1), where high learning engagement emerges from the strategic cultivation of couplings. Furthermore, the profile’s minimal disempowerment scores align with the view that a well-integrated, learner-steered ecosystem may be less susceptible to risks of over-reliance. Collectively, this evidence suggests that AI serves as a pivotal catalyst for high-quality, synergistic couplings among learners, teachers, peers, and the broader learning environment, thereby co-constructing an empowering learning ecosystem.

The Moderate Coupling-Moderate Engagement profile occupies the critical middle ground of functional complementarity and, notably, constituted the largest subgroup (*n* = 228, 47.7%), which is a finding consistent with prior research indicating that moderate engagement levels are prevalent among English learners in China (Li et al., 2022; Xu and Yang, 2024). This profile represents a state of successful but bounded integration: learners demonstrated moderate-to-high levels of AI-ecological coupling and multi-dimensional engagement, confirming functional utility yet falling short of realizing AI’s full synergistic potential. Quantitatively, this was evidenced by robust scores across all interface and engagement measures. Qualitatively, participants (e.g., S6, S7) described AI as being effectively embedded within structured, teacher-guided workflows (e.g., “AI completed the first draft, I revised it myself, the teacher reviewed it…,” S6), characterized by a clear division of labor (“AI served as an aid, the teacher provided guidance…,” S7). This pattern suggests that these learners utilize AI competently for discrete tasks, likely achieving gains in efficiency and support. However, the lack of evidence for metacognitive reconfiguration or pedagogical co-adaptation implies that such use remains within the boundaries of existing learning structures, thereby representing a sub-optimal or non-transformative form of integration where AI augments but does not fundamentally redefine the learning ecology.

This three-part typology thereby resolves the binary narratives prevalent in prior research by demonstrating configural heterogeneity. It directly addresses a key limitation in prior research that frames AI’s impact through a binary lens of “empowerment vs. disempowerment” (Huang et al., 2025; Shi and Aryadoust, 2024; Moorhouse, 2024). By systematically revealing a substantial middle profile and delineating the mechanisms that differentiate all three, our model transcends this dualistic impasse. It demonstrates that heterogeneous outcomes arise from qualitatively distinct configurations of ecological coupling, thereby explaining why the same AI tool can lead to profoundly different learning experiences. The primary differentiator is the learner’s agency in forging coupling quality, not the tool’s inherent features or frequency of use.

Collectively, these profiles form a spectrum of integration, from the structural disconnection of Low Coupling-Low Engagement profile, through the functional complementarity of Moderate Coupling-Moderate Engagement profile, to the orchestration of High Coupling-High Engagement profile. This progression substantiates our central thesis: the primary mechanism driving AI-enabled empowerment is the quality of ecological coupling, not merely the frequency of AI use.

### 4.3 Theoretical contribution: the AI-empowerment Configural Model

**Figure 4:**
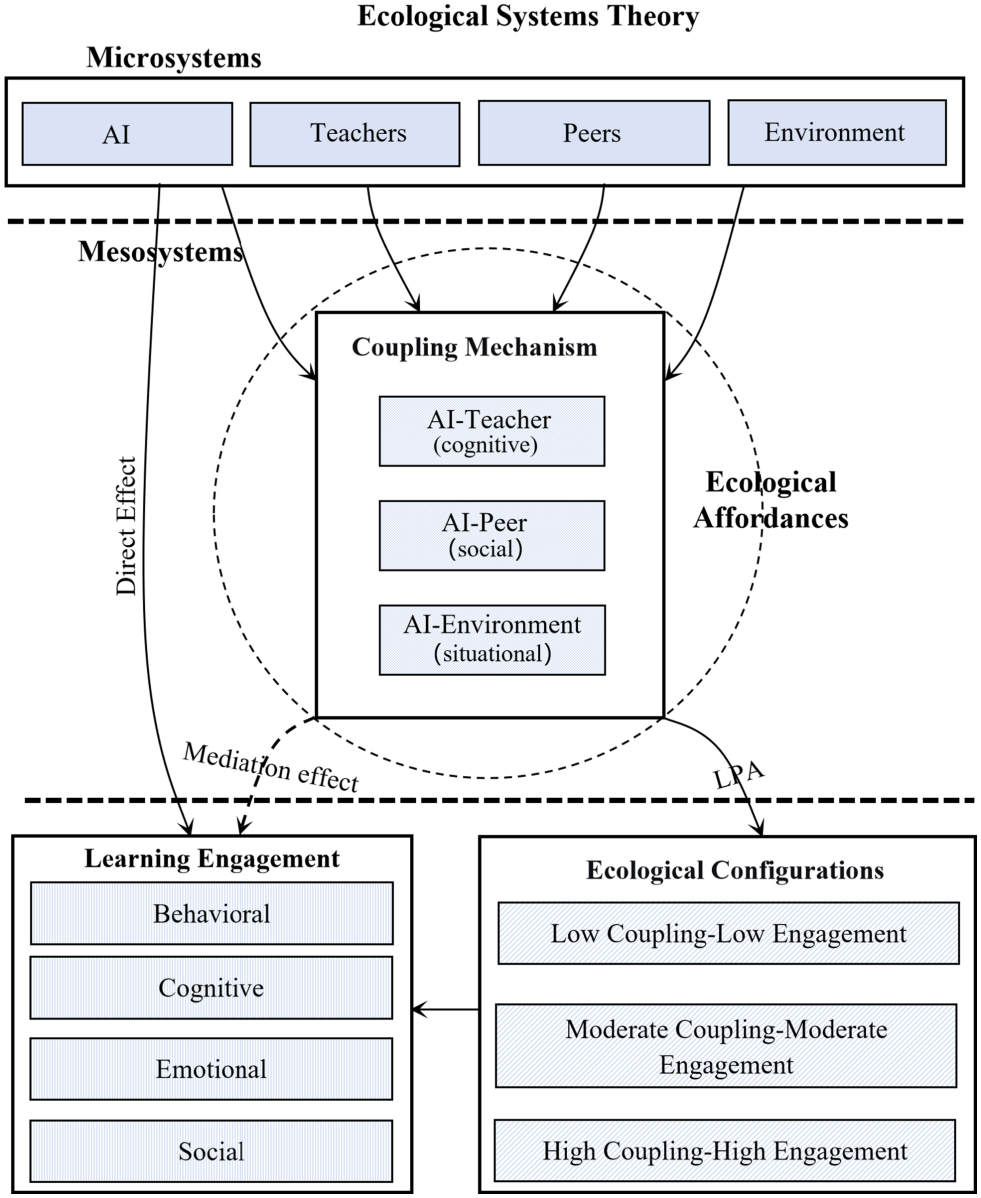
AI-empowerment Configural Model for language learning engagement.

Integrating the mediation pathway (H1) with the configural typology (H2), this study synthesizes an AI-empowerment Configural Model (Figure 4). This model advances existing frameworks by systematically addressing four critical limitations in the current understanding of AI-integrated learning.

#### 4.3.1 Reframing AI: from external tool to internal relational agent

Prevailing ecological perspectives (Jiang, 2025) situate learning within a system of human and contextual elements but often treat technology as an external variable. Our model effects a fundamental conceptual reframing: it reconceptualizes AI as an internal and integral relational agent within the microsystem. This reframing is operationalized and validated through the construct of coupling quality which operationalizes the perceived synergy at three key relational interfaces: AI-Teacher, AI-Peer, and AI-Environment. The finding that the coupling quality, not mere AI use frequency, is the primary mediator of engagement (H1) empirically substantiates this view. Consequently, we theorize AI not as a tool with fixed effects, but as a component whose impact is contingent upon and mediated by the quality of its ecological coupling.

#### 4.3.2 Explaining heterogeneity: from binary narratives to Configural typology

Prior research often presents a binary, contradictory picture of AI as either empowering or disempowering (Karatas et al., 2024; Jiang et al., 2022), lacking a mechanism to explain divergence. The model resolves this impasse by introducing a person-centered configural typology. The three distinct profiles demonstrate that outcomes are not random but arise from qualitatively different holistic configurations of how learners integrate AI. This typology provides a parsimonious explanatory framework for why the same tool yields divergent experiences, moving the field from dualistic narratives to an understanding of the systematic configural heterogeneity.

#### 4.3.3 Integrating levels of analysis: bridging ecosystem structure with learner agency

A persistent theoretical gap exists between macro-structural descriptions of the ecosystem (EST) and micro-perceptual accounts of individual agency (EAT). Our model bridges this gap by synthesizing both levels into a coherent mediated pathway. It posits that the macro-structural integration of AI must be perceived and actualized by the learner as actionable affordances to drive engagement. The significant mediation pathway via perceived coupling quality (H1) operationalizes this bridge, showing how ecosystem structure is subjectively interpreted and translated into learning engagement.

#### 4.3.4 Informing practice: from descriptive labels to diagnostic, actionable categories

Existing technology-user typologies often remain descriptive. However, our model advances practice by grounding its three profiles in a specific explanatory mechanism (coupling quality), transforming them into diagnostic categories. This mechanistic grounding enables precision interventions. For instance, support for the Low Coupling-Low Engagement profile must address fundamental integration failures, whereas support for the Moderate Coupling-Moderate Engagement profile can aim to transcend bounded utility toward synergistic adaptation. Thus, the model shifts guidance from generic tool implementation toward theory-driven, ecological governance.

#### 4.3.5 Theoretical synthesis: integrating EST and EAT for a nuanced ecological account

Ultimately, this work achieves a novel theoretical synthesis by integrating Ecosystem Theory (EST) and Ecological Affordance Theory (EAT). It extends EST by operationalizing the construct of mesosystemic coupling within digital environments, moving beyond metaphor to capture the dynamic quality of AI-microsystem interactions. More profoundly, by integrating EAT, it elucidates the critical link between structure and agency: the potential of the structurally coupled ecosystem is functionally realized only through the learner’s “perception-interpretation-action” cycle (Van Lier, 2004). This synergy decisively shifts the explanatory focus from the intrinsic attributes of the AI tool to the relational dynamics and perceived affordances of the ecosystem itself, providing a more powerful and nuanced account of why identical technological deployments yield profoundly heterogeneous integration outcomes.

### 4.4 Practical implications: from tool implementation to ecological governance

The empirical evidence of this study calls for a fundamental reconceptualization of AI integration in education: from discrete tool implementation to systematic ecological governance. This necessitates viewing AI as an element within ecological learning ecosystems. Accordingly, we propose the following evidence-informed recommendations.

#### 4.4.1 Promoting AI-human coupling through ecological instructional design

The core finding of this study is that AI’s educational impact on learning engagement is significantly mediated by the quality of its ecological coupling. Therefore, instructional design must deliberately engineer high-quality coupling to foster language learning engagement. The following principles demonstrate how coupling design across three interfaces directly translates into enhanced behavioral, cognitive, emotional, and social engagement.

##### 4.4.1.1 Designing AI-teacher cognitive partnerships to elevate cognitive and behavioral engagement

High-quality AI-Teacher coupling creates a cognitive partnership, offloading routine analysis to AI so teachers can focus on high-value mentorship. This design directly boosts cognitive engagement by challenging learners with complex, personalized feedback, and increases behavioral engagement through structured, teacher-facilitated activities. For instance, Shanghai Jiao Tong University’s AI writing evaluator couples with instructors by diagnosing argument logic. Teachers then design targeted workshops. This partnership deepens cognitive engagement as students grapple with higher-order writing concepts, while the clear workshop structure sustains behavioral engagement.

##### 4.4.1.2 Designing AI-peer social scaffolding to foster social and emotional engagement

High-quality AI-Peer coupling acts as social scaffolding, using AI to architect collaborative learning. This design directly cultivates social engagement by structuring productive peer interaction and enhances emotional engagement by reducing isolation and building a sense of community. For instance, at the Open University of China, AI couples individual learning with peer collaboration by forming study groups based on diagnostic data. This scaffolded interaction strengthens social engagement through peer teaching, while the supported collaborative environment promotes positive emotional engagement by mitigating anxiety and fostering belonging.

##### 4.4.1.3 Designing AI-environment contextual integration to enhance emotional and behavioral engagement

High-quality AI-Environment coupling embeds AI as a contextual co-constructor, making it an immersive part of the learning setting. This design heightens emotional engagement through authentic, immersive experiences and increases behavioral engagement by making interactive inquiry within complex environments feasible. For instance, Soochow University’s “Xu Teli” AI assistant couples with the literary-historical context through role-play. This immersion boosts emotional engagement by making learning dramatic and relevant. Similarly, Shenzhen Technology University’s integration of AI as a lab partner couples with the scientific environment, enabling complex experiments that sustain active behavioral engagement.

##### 4.4.1.4 Synthesis: orchestrating holistic coupling for multidimensional engagement

The most transformative outcomes arise from instructional designs that orchestrate holistic coupling across all three interfaces, simultaneously activating multiple dimensions of learning engagement. The University of Edinburgh’s multi-role chatbot exemplifies this integrated approach: it supports teachers (fostering cognitive/behavioral engagement), facilitates peer interaction (enhancing social/emotional engagement), and enriches the learning environment (deepening emotional/behavioral engagement). This ecologically orchestrated design provides a pedagogical blueprint for achieving the High Coupling-High Engagement profile. Ultimately, these cases converge on a core instructional imperative: the pathway to empowered, multidimensional engagement is paved by the intentional design of high-quality ecological coupling across the AI-Teacher, AI-Peer, and AI-Environment interfaces.

#### 4.4.2 Institutional action: cultivating coupling-conducive infrastructures

The finding that ecological coupling quality significantly mediates the relationship between AI use and learning engagement provides a clear mandate for institutional policy: it must shift from merely permitting AI access to proactively constructing infrastructures that nurture such coupling. Given the partial nature of this mediation, a dual-focused policy approach is essential: one that simultaneously encourages the adoption of AI tools and strengthens their deep coupling with human and contextual elements of the learning ecology. To operationalize this, institutions should implement the following concrete measures.

First, establish ecological coupling design criteria. Move beyond evaluating AI tools solely on technical features. Introduce mandatory coupling potential reviews for new technology adoption, assessing: (1) whether the tool’s outputs can naturally feed into peer discussion; (2) whether it provides actionable data for teachers to inform feedback; and (3) how it adapts to or enhances the specific learning context.

Second, develop faculty capacity in “ecosystem design.” Professional development must pivot from “how to use” to “how to integrate.” Training should equip educators to architect learning activities where AI handles foundational tasks, peers engage in scaffolded discussion using AI outputs, and teachers provide higher-order mentorship as well as orchestrating the three couplings simultaneously. Communities of practice should co-design “coupled lesson plans” to create a pedagogical blueprint that facilitates the development of the High Coupling-High Engagement profile.

Third, provide context-sensitive integration frameworks. To prevent the ecological decoupling seen in the Low Coupling-Low Engagement profile, institutions must support contextual adaptation. This involves creating discipline-specific guidelines that demonstrate how to tailor AI tools to local curricular goals and pedagogical traditions. By empowering educators to adapt rather than merely adopt, these frameworks ensure integration is meaningful and mitigate the risks of superficial use and disengagement. Together, these policies translate the core theoretical insight of this study into a scalable institutional action plan to cultivate coupling-conducive infrastructures.

Collectively, these actions institutionalize ecological coupling as a core governance principle. By establishing ecological coupling design criteria, building ecosystem design capacity, and enabling contextual adaptation, institutions shift from managing technology implementation to governing learning ecology, thereby directly activating the mediating pathway identified in this study.

#### 4.4.3 Differentiated support: precision interventions targeting the mediating pathway of coupling quality

The established typology demonstrates that AI’s effect on learning engagement is configural, mediated by coupling quality. Therefore, effective support must be profile-specific. Each profile occupies a distinct position on the mediation pathway, defined by its perceived quality of ecological coupling. Interventions should therefore be designed to directly target and modulate this core perceptual variable.

##### 4.4.3.1 For the low coupling-low engagement profile: interrupting the disconnection cycle with scaffolded re-coupling

This profile is trapped in a cycle where weak perceived coupling leads to disengagement or abandonment. Intervention must forcefully interrupt this cycle by engineering positive, low-threshold coupling experiences. Instruction should provide highly scaffolded tasks where AI’s output is strictly framed as an input for peer or teacher interaction. This design artificially but decisively creates perceived coupling, providing the initial evidence required to activate the dormant mediation pathway.

##### 4.4.3.2 For the moderate coupling-moderate engagement profile: from tool efficiency to strategic orchestration

These learners have activated the mediation pathway but remain within its lower bounds, achieving efficiency without high-quality coupling. The goal is to elevate their position on the pathway through deliberate ecological orchestration. This involves moving beyond using AI for tasks to customizing its role and designing multi-agent learning sequences that intentionally coordinate interactions between AI, self, peer, and teacher resources. This process fosters the meta-cognitive awareness of coupling necessary to transition from bounded utility to transformative co-adaptation.

##### 4.4.3.3 For the high coupling-high engagement profile: leveraging expertise to propagate the pathway

This profile embodies the fully realized mediation pathway, where high coupling quality drives high engagement. They are not merely end-users but exemplars and architects of effective coupling. Institutions should formally institutionalize their expertise by embedding them as peer mentors in structured programs and co-designers of curricular activities. This does more than provide support; it operationalizes them as vectors for disseminating high-quality coupling configurations, thereby strengthening the mediation pathway’s efficacy across the entire learning ecosystem.

##### 4.4.3.4 Aligning assessment with the coupling paradigm

To reinforce these interventions, assessment must shift from evaluating endpoints to diagnosing the coupling process. It should reward coupling-aware artifacts, such as reflective critiques comparing AI and human feedback, or process narratives documenting how learners navigated and integrated inputs from multiple ecological elements. This alignment ensures that assessment itself provides formative feedback on the very dimension the interventions target: the development of coupling quality.

## 5 Conclusion

### 5.1 Research findings

This study establishes that the relationship between AI use and language learning engagement is significantly mediated by learners’ perceived quality of ecological coupling with teachers, peers, and the learning environment. Through LPA, we identified three distinct learner configurations, which demonstrate heterogeneous patterns of AI integration within learning ecosystems. Synthesizing these results, we propose the AI-empowerment Configural Model. This model advances ecological learning theory by reconceptualizing AI not as an isolated tool, but as a relational agent that actively shapes and is shaped by the dynamics of the learning ecology.

### 5.2 Research limitations

This study is subject to several methodological and contextual limitations that warrant careful acknowledgment and qualify the interpretation of its findings.

First, the cross-sectional design precludes definitive causal inferences regarding the proposed mediation pathway from AI use to learning engagement via ecological coupling. While the data are consistent with our theoretical model, the directionality and causality of the relationships remain inferential.

Second, a key constraint lies in the operationalization of AI use as a single-item measure of frequency. Although a pragmatic indicator for initiating ecological activity, this coarse-grained measure fails to capture critical qualitative dimensions such as diversity of purpose, strategic depth, and cognitive engagement quality. This conceptual narrowness may have attenuated the observed effect of AI use on ecological coupling quality, and thus limited a more nuanced exploration of the conditions under which AI fosters ecological integration.

Third, the measurement scales, while developed through a rigorous theoretical and practical process and validated with the main study sample, were not subjected to an independent psychometric pilot test prior to the main survey. The absence of this preliminary optimization step may affect the optimal item performance, factorial robustness, and ultimately the precision of the constructs measuring ecological coupling and engagement.

Finally, the study’s methodological scope is constrained by its exclusive reliance on perceptual, self-report data. The lack of objective behavioral data or observational records restricts our capacity to triangulate findings and to provide a concrete, behavioral account of how ecological coupling is enacted in authentic learning interactions beyond participants’ subjective perceptions.

### 5.3 Future research directions

Directly addressing these limitations, we propose the following four targeted directions for future inquiry to refine the ecological coupling model and enhance its practical utility.

First, future research should employ longitudinal or experimental designs to test the causal mechanisms postulated in our model, moving from correlation to causation.

Second, it is imperative to move beyond AI use frequency. Future studies must develop and validate a multidimensional framework of AI-mediated learning engagement, capturing its essential qualitative facets, such as diversity of application, strategic patterns of AI use, and depth of cognitive interaction. This refined measurement is crucial for identifying which specific configurations of AI use most effectively initiate and strengthen high-quality ecological coupling.

Third, to enhance measurement precision and robustness, future scale development in this domain must include an independent psychometric pilot study prior to large-scale administration. This step is critical for optimizing item wording, verifying factorial structures, and ensuring the reliability and validity of instruments designed for complex ecological constructs.

Finally, future research should systematically integrate multimethod data sources, complementing self-reports with behavioral data from learning analytics, digital traces, or systematic observations. This approach will enable objective triangulation, providing a more comprehensive and concrete understanding of how ecological coupling manifests and functions in real-world learning environments.

## Data Availability

The original contributions presented in the study are included in the article/supplementary material, further inquiries can be directed to the corresponding author.

## Appendix: Computation of effect size metrics

Standardized Indirect Effect (*β*_ab_): Calculated using the formula: *β*_ab_ = (a × b)/*SD_Y_*, where *a* and *b* are the unstandardized path coefficients, and *SD_Y_* is the standard deviation of the dependent variable (Learning Engagement, SD = 0.76,). Thus, 0.056/0.76 ≈ 0.092.

Proportion Mediated (PM): Calculated as the indirect effect divided by the total effect: PM = (a × b)/c = 0.056/0.134 = 0.4179 (41.79%).

Cohen’s *f^2^*: Calculated for each path in the model using the formula: *f*^2^ = *R*^2^*_included_* − *R*^2^*_excluded_*/(1 − *R*^2^*_excluded_*). For Path b (M → Y), this was derived from the change in R^2^ when coupling quality was added to the model predicting engagement, after controlling for AI use frequency.
